# Improving Provider Adoption With Adaptive Clinical Decision Support Surveillance: An Observational Study

**DOI:** 10.2196/10245

**Published:** 2019-02-20

**Authors:** Sundas Khan, Safiya Richardson, Andrew Liu, Vinodh Mechery, Lauren McCullagh, Andy Schachter, Salvatore Pardo, Thomas McGinn

**Affiliations:** 1 Donald and Barbara Zucker School of Medicine at Hofstra/Northwell Manhasset, NY United States; 2 Donald and Barbara Zucker School of Medicine at Hofstra/Northwell Hempstead, NY United States; 3 Office of Chief Informatics Officer Northwell Health Manhasset, NY United States; 4 Emergency Medicine Donald and Barbara Zucker School of Medicine at Hofstra/Northwell Manhasset, NY United States

**Keywords:** pulmonary embolism, clinical decision support, evidence-based medicine

## Abstract

**Background:**

Successful clinical decision support (CDS) tools can help use evidence-based medicine to effectively improve patient outcomes. However, the impact of these tools has been limited by low provider adoption due to overtriggering, leading to alert fatigue. We developed a tracking mechanism for monitoring trigger (percent of total visits for which the tool triggers) and adoption (percent of completed tools) rates of a complex CDS tool based on the Wells criteria for pulmonary embolism (PE).

**Objective:**

We aimed to monitor and evaluate the adoption and trigger rates of the tool and assess whether ongoing tool modifications would improve adoption rates.

**Methods:**

As part of a larger clinical trial, a CDS tool was developed using the Wells criteria to calculate pretest probability for PE at 2 tertiary centers’ emergency departments (EDs). The tool had multiple triggers: any order for D-dimer, computed tomography (CT) of the chest with intravenous contrast, CT pulmonary angiography (CTPA), ventilation-perfusion scan, or lower extremity Doppler ultrasound. A tracking dashboard was developed using Tableau to monitor real-time trigger and adoption rates. Based on initial low provider adoption rates of the tool, we conducted small focus groups with key ED providers to elicit barriers to tool use. We identified overtriggering of the tool for non-PE-related evaluations and inability to order CT testing for intermediate-risk patients. Thus, the tool was modified to allow CT testing for the intermediate-risk group and not to trigger for CT chest with intravenous contrast orders. A dialogue box, “Are you considering PE for this patient?” was added before the tool triggered to account for CTPAs ordered for aortic dissection evaluation.

**Results:**

In the ED of tertiary center 1, 95,295 patients visited during the academic year. The tool triggered for an average of 509 patients per month (average trigger rate 2036/30,234, 6.73%) before the modifications, reducing to 423 patients per month (average trigger rate 1629/31,361, 5.22%). In the ED of tertiary center 2, 88,956 patients visited during the academic year, with the tool triggering for about 473 patients per month (average trigger rate 1892/29,706, 6.37%) before the modifications and for about 400 per month (average trigger rate 1534/30,006, 5.12%) afterward. The modifications resulted in a significant 4.5- and 3-fold increase in provider adoption rates in tertiary centers 1 and 2, respectively. The modifications increased the average monthly adoption rate from 23.20/360 (6.5%) tools to 81.60/280.20 (29.3%) tools and 46.60/318.80 (14.7%) tools to 111.20/263.40 (42.6%) tools in centers 1 and 2, respectively.

**Conclusions:**

Close postimplementation monitoring of CDS tools may help improve provider adoption. Adaptive modifications based on user feedback may increase targeted CDS with lower trigger rates, reducing alert fatigue and increasing provider adoption. Iterative improvements and a postimplementation monitoring dashboard can significantly improve adoption rates.

## Introduction

As adoption of electronic health records (EHRs) has become increasingly widespread, the potential for electronic clinical decision support (CDS) to improve quality of care has been increasingly recognized [1,2]. CDS uses patient-specific information to make assessments and recommendations to the provider at the point of care [3]. Clinical prediction rules (CPRs) are a form of CDS that function as calculators, using elements of a patient’s history, physical examination, and test results to predict the likelihood of a diagnosis, prognosis, or response to treatment [4]. Using well-validated, evidence-based CPRs, CDS tools have reduced antibiotic prescriptions [5], improved lipid management [6], and reduced overtesting [7,8]. However, the impact of these tools has been limited by poor provider adoption, with rates between 10% and 20% [9].

Nonadherence is one of the biggest challenges to the implementation of a successful CDS [10]. Depending on the type of CDS tool, trigger or alert fatigue is an issue that may invariably lead to provider overrides and dismissals. As reported in a study, a provider may receive, on average, 56 alerts per day and spend 49 minutes per day processing them [11]. As an example, alert overrides may occur for 49%-96% of drug safety alerts [12]. User-centered design of CDS with “smart,” targeted triggering to maximize alert appropriateness may improve provider adoption rates [3].

One of the most well-known [13] and well-validated [14] CPRs is the Wells criteria for assessing pulmonary embolism (PE) risk. The need for this CDS is important because emergency departments (EDs) across United States have drastically increased computed tomography pulmonary angiography (CTPA) use for PE evaluation [15,16]. Evidence to justify this increase in utilization is lacking [17], and it places the patient at unnecessary risk to radiation, contrast-induced nephropathy, and increased health care costs [18]. It has been reported that the CTPA yield, a measurement of efficiency [19], ranges between only 7%-10% in the United States [15], suggesting overutilization of this test. The Wells CPR has the potential to rule out 70%-80% of patients without further testing [13,14] and reduce costs of unnecessary testing. By integrating the Wells CPR into the EHR of the ED, Drescher et al found an associated increase in CTPA yield from 9% to 12% for the diagnosis of PE [20]. Despite this improvement in CTPA yield, Drescher et al reported major resistance from the ED physicians, leading to the eventual removal of the tool [20,21]. The findings emphasize the importance of implementing a PE CDS into the providers’ workflow in a way to maximize usability and acceptance.

Our research team developed an electronic Wells CDS tool based on our previous experience in creating CDS tools at the point of care [3]. The first phase of our project included formative assessment and focus groups to determine providers’ level of interest [22], followed by iterative rounds of usability testing for input on design and content of the tool [23]. Using a new usability process called “sensitivity and specificity trigger analysis,” we found the most sensitive way to trigger the CDS tool with minimal sacrifice to the specificity [24]. This process allowed us to limit inaccurate triggering of the CDS tool and reduce trigger fatigue.

A key element in the postimplementation period is the continuous monitoring and sustainability of the tool among clinical providers [3,24]. It has been noted that evaluations of postimplementation alerts’ appropriateness can be labor-intensive and costly [25]. Nevertheless, investigators are beginning to develop tools to efficiently evaluate alerts [26]. With the launch of our CDS tool based on the Wells criteria for PE, we developed a system to track the trigger rate (defined as the number of times the CDS tool is triggered divided by the number of total visits). Based on analyses of the trigger rates over time and modifications to the CDS, we hypothesized that iterative changes to the Wells CDS can lead to an increase in the adoption of the tool.

## Methods

Our research team consisted of expert evidence-based medicine researchers, implementation scientists, health informaticists, and internal medicine and emergency medicine physicians. We worked with Allscripts’ EHR and Sunrise Emergency Care (Allscripts Healthcare, Llc) to develop and integrate the Wells CDS tool into the EDs of 2 tertiary care centers’ within our health care organization. All study procedures were approved by the Institutional Review Board and the Emergency Medicine Research Committee within Northwell Health.

**Figure 1:**
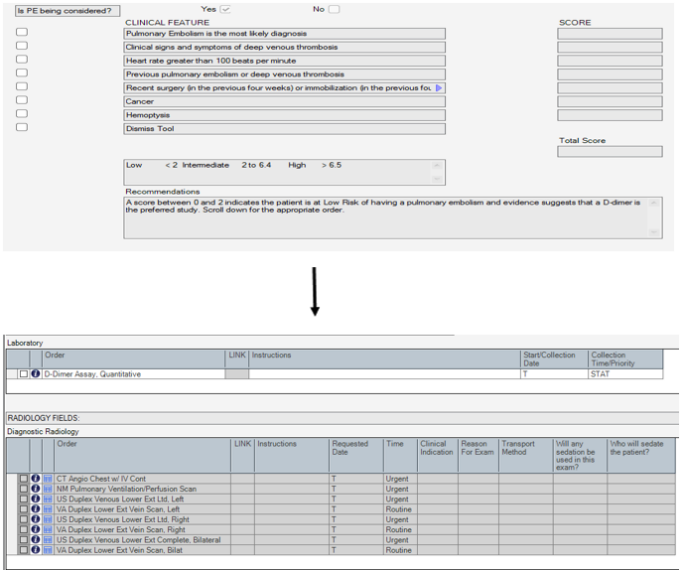
Wells criteria for pulmonary embolism, recommendations, and order set. Source: Allscripts Healthcare Solutions.

The CDS tool developed includes a calculator, with risk factors from the Wells PE clinical rule, and a dialogue box to outline recommendations, with an accompanying order set that illuminates orders according to the risk stratification (Figure 1). After thorough usability testing of the tool in the EHR playground environment within our Usability Lab [22-24], we launched the tool within the emergency rooms at 2 large academic tertiary centers in a staggered rollout. The initial design aspect of the usability testing was analyzing the sensitivity and specificity of triggers that would elicit the Wells CDS tool to trigger [24]. Critical characteristics of the tool development that employ user-centered design include qualitative research (interviews) to learn about users’ context and workflow, usability surveys, system usage data, and “think aloud” interviews outlined by the study team in the usability testing and formative assessment articles [22,23].

When integrated into the order entry workflow, the Wells CDS tool would be triggered when a provider attempted to order any test that is used to evaluate a suspected PE. Initially, the triggers were D-dimer, computed tomography (CT) chest, CT angiography, ventilation-perfusion scan, and lower extremity Doppler ultrasound (Textbox 1). Upon triggering, the provider filled out the Wells CDS tool as it appears in Figure 1. The completed tool calculated the patient’s risk for PE and stratified the patient into low, intermediate, or high categories, each with a bundled order set that the provider could choose for the next step [23].

Clinicians attempting to order CTPA in a low-risk patient were able to order by dismissing the tool, after which all orders become ungrayed and visible. If a patient had a positive D-dimer, the clinician would move forward with ordering the CTPA and would not be forced to order the D-dimer despite the low risk. If a patient was intermediate and high risk, both D-dimer and CTPA were available for the clinician to order. It is under the clinician’s discretion to order the preferred test for these two groups as suggested through current literature [27]. Exclusion criteria for the tool triggering included individuals aged <18 years, as the CDS was firing at 2 tertiary adult hospitals.

**Figure 2:**
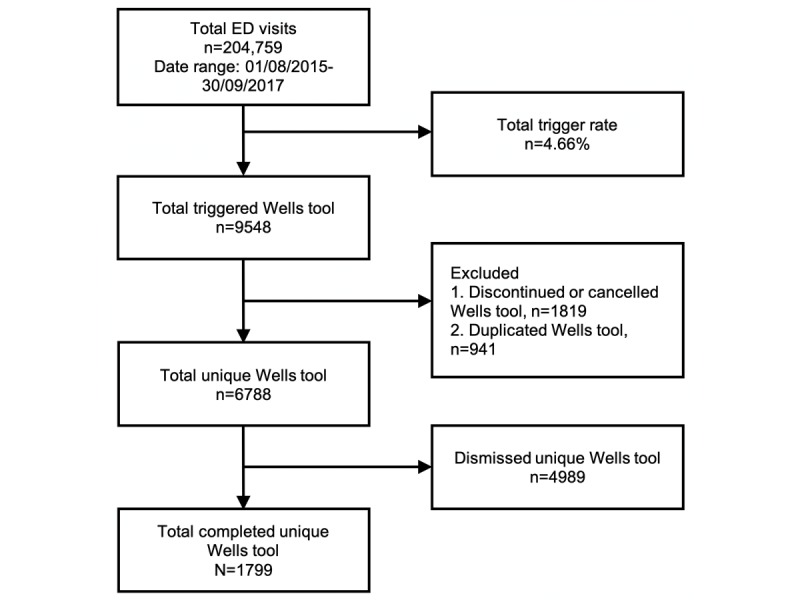
Summary report for the emergency department (ED) of tertiary center 1.

**Figure 3:**
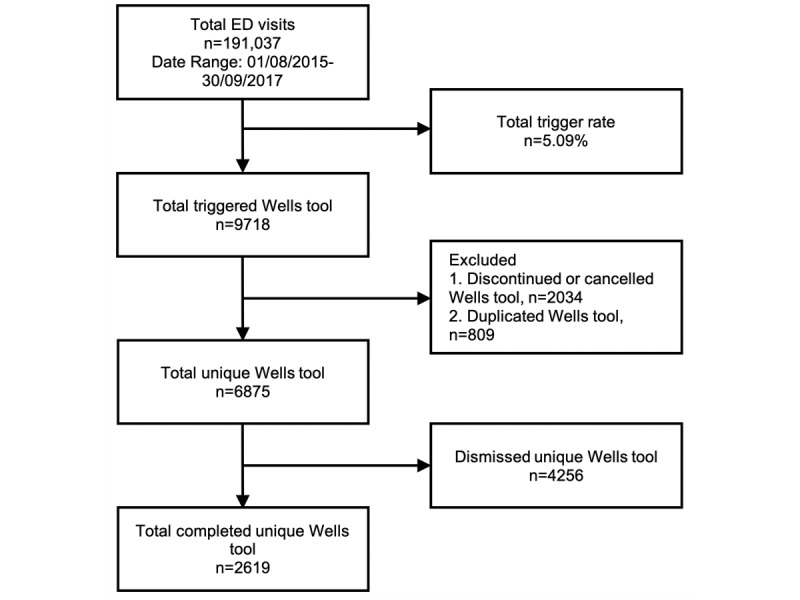
Summary report for the emergency department (ED) of academic tertiary center 2.

A trigger rate tracking tool was developed using Tableau (Tableau Software) to monitor adoption and trigger rates of the Wells CDS tool implementation. This tool gave a monthly status report of each of the 2 tertiary hospitals’ EDs’ usage of the tool by providing the trigger rate by taking the number of times the Wells CDS tool was triggered over the total number of ED visits during the same period. The tracking tool also monitored the number of times the triggered CDS tool was completed, with the provider using the tool to place orders for PE evaluation. This gives us the completion rate of the Wells CDS tool when divided by the total number of triggers opened for that period. Figures 2 and 3 demonstrate the logic for arriving at trigger rates and completion rates for each tertiary center.

**Wells clinical decision support tool trigger points at order entry.**

Computed tomography (CT) angiography chest with contrast

CT chest with contrast

D-dimer

Nuclear medicine pulmonary ventilation-perfusion scan

Ventilation-perfusion scan

Lower extremity Doppler ultrasound

**Figure 4:**
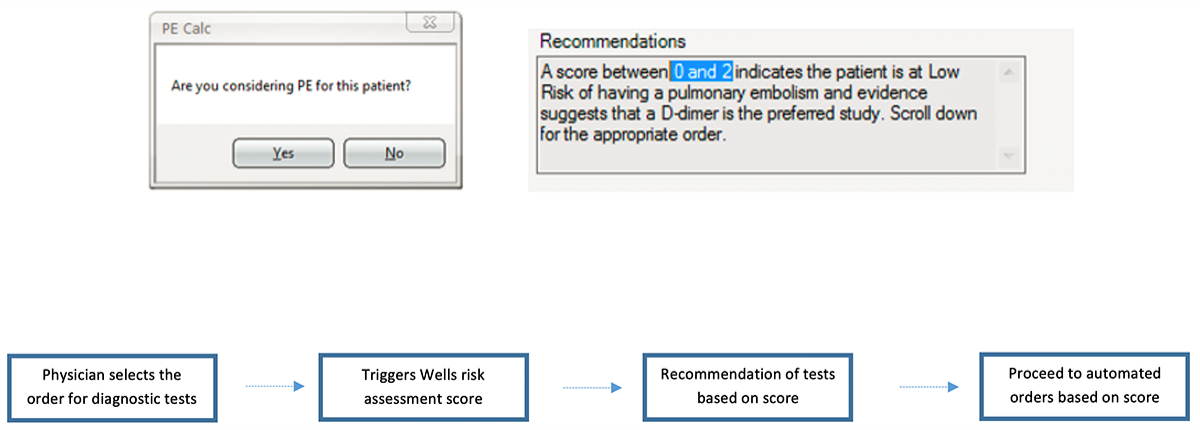
Dialogue box, dynamic label for recommendations, and tool logic. Source: Allscripts Healthcare Solutions.

After deployment of the Wells CDS tool at the 2 tertiary centers, we conducted focus groups with qualitative feedback with ED providers. We synthesized these feedbacks and developed modifications to the CDS. In December 2015, we implemented 3 iterative changes to the Wells CDS tool. First, our Sunrise CDS team removed CT chest as a trigger for the CDS tool. Then, the team updated the PE order availability algorithm, allowing a low risk (Wells score <2) to open up a lab order called “D-Dimer Assay, Quantitative” and allowing the intermediate risk (Wells score 2-6) to open up the order for “Imaging Studies.” Last, a dialogue box was added to appear before the actual order set opened, and a Dynamic Label functionality was added for the “Recommendations” field in the order set (Figure 4). This functionality allowed the actual score (result of documentation) to be referenced in the recommendations field (highlighted) instead of a generic message with static numbers. As outlined in Figure 4, the tool logic was triggered when a clinician entered a diagnostic test for PE that prompted the Wells criteria calculator, recommendations, and accompanying order sets.

## Results

From the time the Wells CDS tool was deployed in the tertiary academic centers (demographic characteristics in Table 1) to the implementation of the CDS modifications, the trigger rates were relatively high for both tertiary centers.

**Figure 5:**
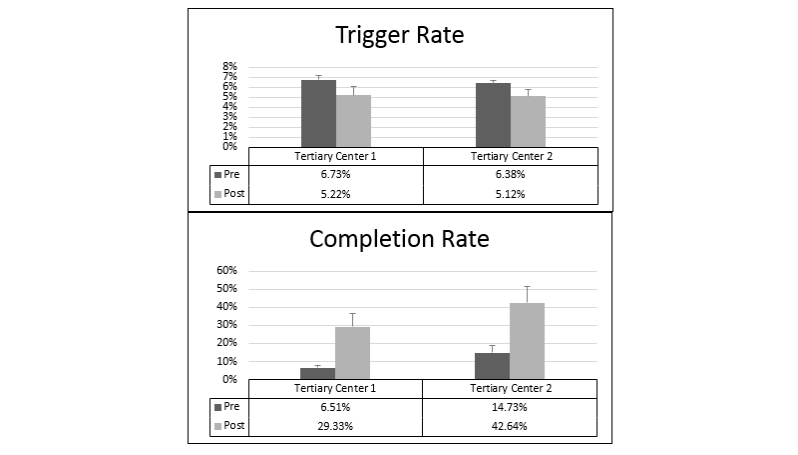
Average trigger and completion rates at tertiary centers 1 and 2 pre- and postmodifications.

As seen in Figure 5, the average trigger rates were 6.73% (2036/30,234 visits; 95% CI 6.33%-7.13%) and 6.37% (1892/29,706 visits; 95% CI 6.06%-6.70%) at tertiary centers 1 and 2, respectively. The average completion (adoption) rates of the tool were relatively low 6.5% (23.20/360; 95% CI 5.08%-7.94%) and 14.7% (46.60/318.80; 95% CI 10.69%-18.77%) for tertiary center 1 and 2, respectively.

In December 2015, modifications to the CDS tool were implemented to optimize the triggering event. The 5-month period after implementation of changes is termed the “postmodification period” here to contrast with the “premodification period.” At tertiary center 1, the average trigger rate decreased to 5.20% (1629/31,361 visits; 95% CI 4.37%-6.07%). Adoption rates increased to 29.3% (81.60/280.20; 95% CI 22.20%-36.46%), a staggering 4.5-fold increase. Similarly, at tertiary center 2, the average trigger rate dropped to 5.11% (1534/30,006 visits; 95% CI 4.51%-5.73%). The adoption rates increased to 42.6% (111.20/263.40; 95% CI 33.56%-51.72%), an almost 3-fold increase.

**Figure 6:**
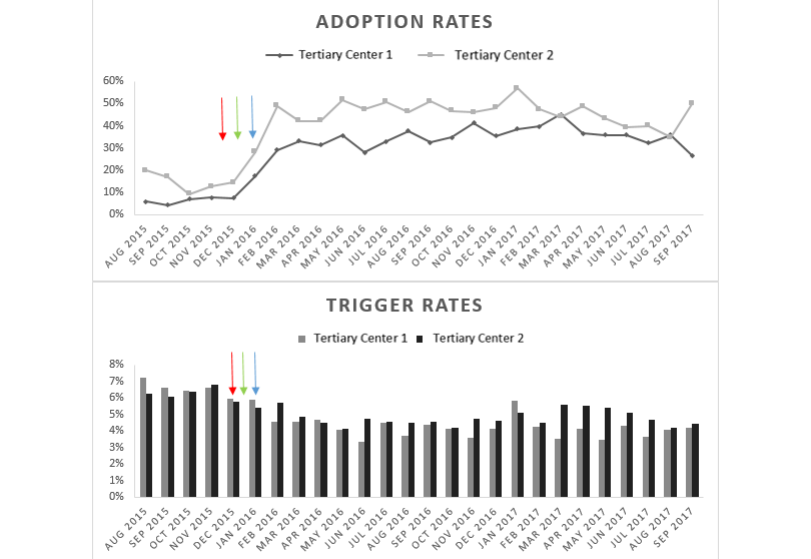
Wells clinical decision support tool adoption and trigger rates at tertiary centers 1 and 2.

The significant increase in adoption rate in 2015 is evident in Figure 6, after the modifications to the Wells CDS tool were implemented. This graph shows the sustainability of the adoption rate upsurge well past the 5-month “postmodification period” we examined above. At the same time, the graph shows a decline in trigger rates after the initial implementation of the CDS tool. In the figure, arrows mark the time of modifications. Red arrow indicates removing CT chest as a trigger, green arrow indicates allowing CTPA for an intermediate score, and blue arrow indicates adding the dialogue box for “Are you considering PE for this patient?” and the dynamic label.

**Table 1:** Demographics of populations in tertiary academic centers.

Race or ethnicity	Patients, n (%)	
	Tertiary center 1 (n=981,701)	Tertiary center 2 (n=498,256)
White	276,181 (28)	287,391 (58)
Hispanic	234,050 (24)	68,864 (14)
African American	153,044 (16)	19,587 (4)
Asian	253,245 (26)	113,444 (23)
Other or multirace	60,085 (6)	8530 (1)

## Discussion

### Principal Findings

The integration of CDS into an EHR to guide management plans has been well documented to improve patient care [5-7]. Use of the Wells criteria for PE increases the yield of CTPA for suspected PE [28]. Unfortunately, as evidenced by Drescher et al’s study, the CDS was poorly accepted by emergency physicians and was eventually removed despite showing positive results [20,21]. It is, thus, crucial to devise computerized tools that are optimally integrated into clinician workflow without causing alert fatigue [24] because usability may be as important as accuracy and effectiveness in implementing CDS [23]. We speculate that the high adoption rates of 29.3% (81.60/280.20) to 42.6% (111.20/263.40) observed in our study, as opposed to an average of 10%-20% adoption rates from previous CDS research [9], were a result of the comprehensive user-centered development process including workflow assessment of the ED, focus groups, usability testing, and collaboration with a multidisciplinary team [22-24].

We developed an electronic Wells CDS tool based on user-centered design principles from our extensive experience. As our past research study has shown, usability testing prior to integration of the tool can lead to high adoption rates [3]. Thus, we implemented a two-phase usability testing approach with emergency physicians prior to the integration of the Wells CPR into the EHR [23]. Despite rounds of usability testing, in a near-live environment, where multiple triggers were created, the use of CT chest with contrast as a trigger was an oversight and was not picked up. The near-live environment is able to mimic the live environment to a certain degree of limitation, which further reiterates the importance of postimplementation monitoring and evaluation of CDS use.

Furthermore, the sensitivity and specificity trigger analysis was developed to identify optimal trigger locations to avoid underutilization and overtriggering [24]. From our past studies, we have also learned that adoption of CDS tools can be dependent on individual user characteristics such as age, training level, and experiences with health technology [29]. It was found that while attendings had the most experience with CDS tools, they were least likely to use them compared with the residents [29]. A dynamic and adaptive design may have a large impact on the adoption of CDS tools [30]. To monitor and prepare for an adaptive electronic CDS with iterative changes, we designed a new tracking method for trigger rate.

A key concept behind developing the tracking mechanism and monitoring adoption rates is maintenance, which is one of the essential pieces of the RE-AIM theoretical framework. The framework is utilized to understand translational and system-change efforts in health care [31]; the different dimensions of the theory include research, effectiveness or efficacy, adoption, implementation, and maintenance. The theory has been used in several studies to successfully provide evidence that help informatics interventions overcome their frequent deficiencies in external validity [32-34]. Following implementation of the Wells CDS, we obtained feedback from the end users in accordance with the theoretical framework to further assess adoption and implementation. A key aspect of the framework is maintenance, and as the study team continued to monitor the uptake of the Well’s CDS tool and review current literature, the appropriate addition to include imaging as an option for intermediate-risk patients was implemented.

The postimplementation tracking method trends the trigger rate and completion rate of the Wells CDS tool at our 2 tertiary hospitals. The combination of these rates gives us the sense of the CDS acceptability and adoption on a monthly basis. We have demonstrated that postimplementation tracking and iterative changes to a deployed CDS can result in a 4.5-fold increase in adoption rate. Similar mechanisms have been implemented to track the effectiveness of CDS; Chaffee et al devised monthly reports on alert occurrence and override rates organized into a dashboard view [35]. Using postimplementation tracking methods such as this allows for the development of novel metrics for predicting inappropriate alerts and responses [25]. A “smart” alert system can be used to track a clinician’s response to a specific alert and identify inappropriate alerts [25].

As far as we know, this is the first reported use of a postimplementation tracking mechanism to monitor Wells CDS tool adoption rate. Prior studies have recognized the potential and effectiveness of CDS if properly implemented and utilized. While usability testing and a user-centered design process have helped improve initial provider uptake, longitudinal studies have shown a decrease in user participation as time goes on [36]. The development of a dynamic and adaptive CDS may help improve and sustain the adoption rate. This postimplementation CDS tracking method may serve as a springboard for the study and design of a “smart” CDS down the line.

### Limitations

The user preferences may vary depending on the institution, and willingness to complete the tool will depend on individual workflows and cultural norms. Providers participating in the study were mostly residents and attending physicians. We would like to incorporate triage nurses in the next assessment with an upstream triage alert for the CDS.

Additional results of the ongoing study are forthcoming and will discuss ordering behavior, adoption by the provider, and overall effectiveness of the CDS tool in the evaluation of PE, which have not been outlined in the Discussion section.

The study was limited due to a single-institution setting, which may limit generalizability. However, the study included 2 academic tertiary centers within a large health system, with vastly different demographics to alleviate this constraint.

### Conclusions

Implementation of electronic CDS has shown to improve patient outcomes. However, overtriggering or alerting of the CDS may lead to provider nonadherence and poor adoption of the tool. Postimplementation evaluation of the CDS trigger rate and adaptive modifications of the triggers may lead to more targeted triggers and improvements in the CDS adoption rate. This study provides an example of how iterative changes and postimplementation tracking mechanism of the CDS result in a significantly improved adoption rate.

## Abbreviations

CDS: clinical decision support
CPR: clinical prediction rule
CT: computed tomography
CTPA: computed tomography pulmonary angiography
ED: emergency department
EHR: electronic health record
PE: pulmonary embolism
